# Transactions of the Medical Society, of the State of New York

**Published:** 1879-04

**Authors:** 


*Transactions of the Medical Society of the State of New York.* This volume is reduced in dimensions compared with former issues. Though we cannot say is in any degree less valuable. It is or should be in the hands of all physicians of the State, and consequently requires no detailed mention of contents.

